# Interleukin-9 regulates macrophage activation in the progressive multiple sclerosis brain

**DOI:** 10.1186/s12974-020-01770-z

**Published:** 2020-05-06

**Authors:** Gloria Donninelli, Inbar Saraf-Sinik, Valentina Mazziotti, Alessia Capone, Maria Grazia Grasso, Luca Battistini, Richard Reynolds, Roberta Magliozzi, Elisabetta Volpe

**Affiliations:** 1 Neuroimmunology Unit, IRCCS Fondazione Santa Lucia, Via del Fosso di Fiorano 64, 00143 Rome, Italy; 2 Department of Neurobiology, Weizmann Institute of Science, Rehovot, Israel; 3 Neurology section, Department of Neurosciences, Biomedicine and Movement Sciences, University of Verona, Policlinico G.B. Rossi, P.le L.A. Scuro, 10, 37134 Verona, Italy; 4 Department of Biology and Biotechnology Charles Darwin, Sapienza University, Rome, Italy; 5 Multiple Sclerosis Centre, IRCCS Fondazione Santa Lucia, Rome, Italy; 6 Division of Neuroscience, Department of Brain Sciences, Imperial College London, London, UK

**Keywords:** Progressive multiple sclerosis, Interleukin-9, Interleukin-9 receptor, Macrophages, Inflammation

## Abstract

**Background:**

Multiple sclerosis (MS) is an immune-mediated, chronic inflammatory, and demyelinating disease of the central nervous system (CNS). Several cytokines are thought to be involved in the regulation of MS pathogenesis. We recently identified interleukin (IL)-9 as a cytokine reducing inflammation and protecting from neurodegeneration in relapsing–remitting MS patients. However, the expression of IL-9 in CNS, and the mechanisms underlying the effect of IL-9 on CNS infiltrating immune cells have never been investigated.

**Methods:**

To address this question, we first analyzed the expression levels of IL-9 in post-mortem cerebrospinal fluid of MS patients and the in situ expression of IL-9 in post-mortem MS brain samples by immunohistochemistry. A complementary investigation focused on identifying which immune cells express IL-9 receptor (IL-9R) by flow cytometry, western blot, and immunohistochemistry. Finally, we explored the effect of IL-9 on IL-9-responsive cells, analyzing the induced signaling pathways and functional properties.

**Results:**

We found that macrophages, microglia, and CD4 T lymphocytes were the cells expressing the highest levels of IL-9 in the MS brain. Of the immune cells circulating in the blood, monocytes/macrophages were the most responsive to IL-9. We validated the expression of IL-9R by macrophages/microglia in post-mortem brain sections of MS patients. IL-9 induced activation of signal transducer and activator of transcription (STAT)1, STAT3, and STAT5 and reduced the expression of activation markers, such as CD45, CD14, CD68, and CD11b in inflammatory macrophages stimulated in vitro with lipopolysaccharide and interferon (IFN)-γ. Similarly, in situ the number of activated CD68^+^ macrophages was significantly reduced in areas with high levels of IL-9. Moreover, in the same conditions, IL-9 increased the secretion of the anti-inflammatory cytokine, transforming growth factor (TGF)-β.

**Conclusions:**

These results reveal a new cytokine expressed in the CNS, with a role in the context of MS. We have demonstrated that IL-9 and its receptor are both expressed in CNS. Moreover, we found that IL-9 decreases the activation state and promotes the anti-inflammatory properties of human macrophages. This mechanism may contribute to the beneficial effects of IL-9 that are observed in MS, and may be therapeutically potentiated by modulating IL-9 expression in MS.

## Background

Interleukin (IL)-9 is a cytokine involved in the development of allergic response and in immune responses against intestinal nematodes [1, 2]. The involvement of IL-9 in human autoimmunity has been studied in psoriasis, where it was shown that IL-9 receptor (IL-9R) is increased in lesioned compared to healthy skin [3], and in lupus erythematous, where IL-9 was reported to be highly expressed in the serum [4]. We recently demonstrated an important immunoregulatory role of IL-9 in relapsing–remitting (RR) multiple sclerosis (MS), where the expression levels in the cerebrospinal fluid (CSF) of RR-MS patients inversely correlate with indexes of inflammatory activity, neurodegeneration, and disability progression of MS [5]. Here, we characterize, for the first time, the expression of IL-9 in the central nervous system (CNS) of secondary progressive MS patients.

IL-9-induced cell responses are mediated by the IL-9R. This heterodimeric receptor is composed of a specific alpha chain and a gamma chain that it shares with the IL-2 receptor. The binding of IL-9 with its receptor promotes cross-phosphorylation of Janus kinase (JAK) 1 [6] and JAK3 [6], thus leading to activation of signal transducer and activator of transcription (STAT) 1, 3, and 5 [7].

The downstream effects of IL-9 have been primarily described in mast cells in the context of allergic responses. However, the effect of IL-9 on other immune cell types and under different contexts is yet to be studied. In fact, a systematic analysis of IL-9R expression among blood cells has never been performed. Here, we conduct a comparative study of IL-9R expression by the different immune cell types composing the human blood. Our analysis reveals that monocytes express the highest levels of IL-9R, suggesting they are the most responsive immune cells to IL-9. Accordingly, previous studies demonstrated that human monocytes and alveolar macrophages are affected by IL-9. Specifically, these studies demonstrated that IL-9 reduced the respiratory burst and inflammatory cytokine release [8, 9].

However, the role of IL-9 on resident macrophages or those infiltrating the CNS has never been investigated. It is known that under physiological conditions, resident microglia is involved in host defense, immune regulation, tissue homeostasis, and regeneration [10, 11]. Upon inflammatory or pathological injury, blood monocyte-derived macrophages infiltrate the CNS and support microglia in driving and controlling the immune responses. Thus, under chronic CNS inflammation, macrophages become particularly relevant, and dysregulation of macrophage functions may underlay the pathogenesis of many CNS diseases, including MS.

Stimulated macrophages adopt context-dependent phenotypes that either promote or inhibit inflammatory responses. In fact, depending on the microenvironmental signals of the local milieu, macrophages show a spectrum of activated phenotypes that in vitro could be simulated by using pro-inflammatory and anti-inflammatory polarization assay [12, 13].

Thus, microglia/macrophages are multifunctional cells, and the dynamic balance between pro- and anti-inflammatory functions is crucial in MS and its mouse model: experimental autoimmune encephalomyelitis (EAE). The disturbance of this balance has a key role in lesion pathogenesis and activity, and therefore in disease progression [14].

Our study revealed that IL-9, expressed in active lesions of MS, might modulate inflammatory macrophage properties in the CNS. Collectively these results could lead to novel therapeutic strategies aimed at reverting the pathogenic macrophage dysregulation in MS.

## Methods

### Neuropathology study on post-mortem MS brain samples

#### Immunohistochemistry and immunofluorescence

Snap frozen tissue blocks from 10 post-mortem brains of secondary progressive MS patients and of 3 post-mortem controls with non-inflammatory neurological conditions were analyzed. The brains were obtained in autopsy at the UK MS Society Tissue Bank at Imperial College, under ethical approval by the National Research Ethics Committee (08/MRE09/31). Serial fresh sections (10-μm thick) were immunostained with myelin oligodendrocyte glycoprotein (MOG) or major histocompatibility complex (MHC) class II antibodies for neuropathological assessment of lesion presence and activity following previously published procedures [15]. In addition, to validate the activity stage of the lesion, double staining with MHC class II and MOG antibodies was also performed. Serial frozen sections, cut from the same blocks, were stained using a specific anti-IL-9 rabbit polyclonal antibody or anti-IL-9R mouse monoclonal antibody. We performed double immunohistochemistry (IHC) and immunofluorescence (IF) staining, combining antibodies specific for IL-9 and markers CD68, TMEM119, and MHC class II. For MOG, IL-9R, CD68 immunostaining treatment with cold methanol was performed, whereas for HLA (DP, DQ, DR), IL-9 and TMEM119 was used ethanol. Details of the antibodies used for IHC and IF are reported in Table 1. Images, were acquired using an Axiophot microscope (Carl Zeiss, Jena), equipped with a digital camera (Axiocam MRC) and analyzed using the Axiovision 6 AC software.

**Table 1:** Antibodies used for immunostainings

Primary antibody	Fluorophore	Origin	Target	Dilution	Source	Application
CX3CR1	FITC	Rat (mAB; IgG2b)	Chemokine C-X3-C receptor 1	1:160	Biolegend, California, USA	FC
CD206	PE	Mouse (mAB; IgG1)	Mannose receptor	1:50	Beckman Coulter, California, USA	FC
HLA-DP, DQ, DR	BV785	Mouse (mAB; IgG2a)	MHC class II antigen	1:100	BD Bioscience	FC
CD11b	PerCP-Cy5.5	Mouse (mAB; IgG1)	Alpha M integrin	1:80	Biolegend, California, USA	FC
CD45	PE-Cy7	Mouse (mAB; IgG)	Leukocyte common antigen	1:100	Beckman Coulter, California, USA	FC
CD68	APC	Mouse (mAB; IgG2b)	CD68 110-kDa transmembrane glycoprotein	1:20	Miltenyi, Germany	FC
CD14	APC-eFluor780	Mouse (mAB; IgG1)	Leucine-rich repeat (LRR) protein	1:100	eBioscience	FC
CD163	VioBlue	Mouse (mAB; IgG1)	Hemoglobin-haptoglobin scavenger receptor	1:100	Miltenyi, Germany	FC
IL-9R	BV421	Mouse (mAB; IgG2b,k)	Interleukin-9 receptor alpha	1:30	BD Bioscience	FC
IL-9R	PE	Mouse (mAB; IgG1)	Interleukin-9 receptor alpha	1:20	R&D Systems, Minnesota, USA	FC
IgG1 Isotype	PE	Mouse	–	1:20	R&D Systems, Minnesota, USA	FC
IgG2b,k Isotype	BV421	Mouse	–	1:30	BD Bioscience	FC
MOG	–	Rabbit (pAB; IgG)	Myelin oligodendrocyte glycoprotein	1:50	Proteintech, Manchester, UK	IHC
HLA-DP, DQ, DR	–	Mouse (mAB; IgG1)	MHC class II antigen	1:50	Dako, Glostrup, Denmark	IHC
CD68	–	Mouse (mAB; IgG1)	CD68 110-kDa transmembrane glycoprotein	1:25	Dako, Glostrup, Denmark	IHC
TMEM 119	–	Rabbit (pAB; IgG)	Transmembrane protein 119	1:50	Atlas Antibodies, Bromma, Sweden	IHC
IL-9	–	Rabbit	Interleukin-9	1:50	Santa Cruz Biotechnology, INC.	IHC/IF
IL-9R	–	Mouse (mAB; IgG)	Interleukin-9 receptor	1:50	MAB290; R&D Systems	IHC
CD4	–	Mouse (mAB; IgG1)	CD4^+^ T lymphocytes	1:50	ThermoFisher, Rockford, USA	IF
CD8	–	Mouse (mAB; IgG1)	CD8^+^ T lymphocytes	1:50	Dako, Glostrup, Denmark	IF

List of antibodies used for immunohistochemistry, immunofluorescence, and flow cytometry

#### Analysis of IL-9 production

CSF samples from 29 post-mortem MS patients (age 60, 2 ± 18,8; mean ± SD) and 17 age-matched controls (age 62, 6 ± 17,2; mean ± SD) with other neurological diseases were centrifuged to eliminate cells and cellular debris and immediately stored at − 80 °C until analyzed. All samples were processed using identical standardized procedures using a Bio-Plex Multiplex Cytokine Assay (Bio-Rad Laboratories), according to the procedures previously optimized [16]. Concentrations of IL-9 were calculated according to a standard curve generated for each target and expressed as pg/ml. When the concentrations of the cytokines were below the detection threshold, they were assumed to be 0 pg/ml.

### In vitro study

#### Purification of monocytes from blood and in vitro differentiation of macrophages

Peripheral blood mononuclear cells (PBMCs) were purified from buffy coats of healthy adult (range 20–65 years of age) volunteer blood donors (independently of sex), and three patients with RR-MS according to established criteria [17] by density gradient over Ficoll-Hypaque (Amersham Pharmacia Biotech, Uppsala, Sweden).

Approval by the ethics committee of the Fondazione Santa Lucia and written informed consent in accordance with the Declaration of Helsinki from all participants were obtained before study initiation.

CD14^+^ monocytes were purified by immunomagnetic depletion using Easy Sep human monocyte enrichment kit (Stem Cell). Purified monocytes CD14^+^ showed a purity > 96%, as revealed by flow cytometry analysis (data not shown). Monocytes were cultured at a density of 500,000 cells/ml in 6-well plates (Falcon) in RPMI 1640 with 10% of Fetal Bovine Serum (FBS) and induced to differentiate toward macrophages for 3 days in the presence of M-CSF (50 ng/ml). At day 3 and 6, half of the medium was changed with fresh medium and M-CSF (25 ng/ml). At the end of the culture, the cells were pre-incubated or not for 24 hours (h) with IL-9 (20 ng/ml), before being cultured for further 48 h in the absence of stimuli, or in the presence of Lipopolysaccharides (LPS) (10 ng/ml) and Interferon (IFN)-γ (50 ng/ml) to obtain pro-inflammatory macrophage subtype [18].

#### Flow cytometry analysis

Receptors expressed by macrophages were analyzed by staining with mouse anti-human CX3CR1 (FITC), CD206 (PE), HLA-DR (BrilliantViolet 785), CD163 (VioBlue), CD11b (PerCP-Cy5.5), CD45 (PE-Cy7), CD68 (APC), CD14 (APC-eFluor780), IL-9R Subunit alpha (PE or BrilliantViolet 421), Mouse IgG1 Isotype control (PE), and Mouse IgG2b,k Isotype control (BrilliantViolet 421), with Fixable Aqua Dead Cell Stain (Thermo Fisher Scientific). Details of the antibodies used for flow cytometry (FC) are reported in Table 1. Samples were analyzed on a Cytoflex (Beckman Coulter), and data were analyzed with FlowJo (Tree Star, Ashland, OR, USA).

#### Analysis of cytokine production

IL-9, IL-6, TGF-β, and cytokines in culture supernatants were measured by ELISA kits (eBioscience), IL-10 was measured by ELISA kit (RnD Biosystems), IL-12 was measured by a high sensitivity ELISA kit (RnD Biosystems), IL-27 was measured by ELISA kit (My Biosource), and IL-35 was measured by ELISA kit (Cloud-Clone Corporation), according to manufacturer’s instructions.

#### Western blot analysis

Protein extraction was performed as previously described [5]. Membranes were incubated with the following antibodies overnight at 4 °C: rabbit polyclonal anti-human phospho-STAT1 (Y701) (Cell Signaling; 1:1000 dilution), rabbit polyclonal anti-human phospho-STAT3 (Y705) (Cell Signaling; 1:1000 dilution), mouse IgG1 anti-human phospho-STAT5 (Y694) (BD Transduction Laboratories; 1:500 dilution), rabbit polyclonal anti- human IL-9R (Abcam; 1:500 dilution), rabbit anti-human alpha/beta tubulin (Cell Signaling; 1:1000 dilution), and mouse anti-human actin (Abgent; 1:3000 dilution). All antibodies were diluted in 3% non-fat dry milk in PBS, containing 0.1% Tween-20. Secondary anti-mouse or anti-rabbit IgGs conjugated to horseradish peroxidase (Cell Signaling) were incubated with the membranes for 1 h at room temperature at a 1:2000–1:3000 dilution in PBS containing 3% non-fat dry milk and 0.1% Tween 20. Immunostained bands were detected by chemiluminescent method (SuperSignal West Pico PLUS ECL Substrate, Thermo Scientific).

### Statistical analysis

For pair-wise comparisons of different conditions from the same donors or different donors, we used a non-parametric two-tailed paired or unpaired *t* test, respectively. Two-way Analysis of Variance (ANOVA) was performed to analyze the main effects of two conditions on the dependent variables and their interactions. Data were presented as mean ± standard error (SEM). The Pearson correlation coefficient was used to assess the significance of correlation among the count of CD68^+^ and IL-9^+^ cells. A *p* value (*p*) of less than 0.05 was considered statistically significant.

## Results

### IL-9 is expressed in the central nervous system of secondary progressive MS patients

**Fig. 1:**
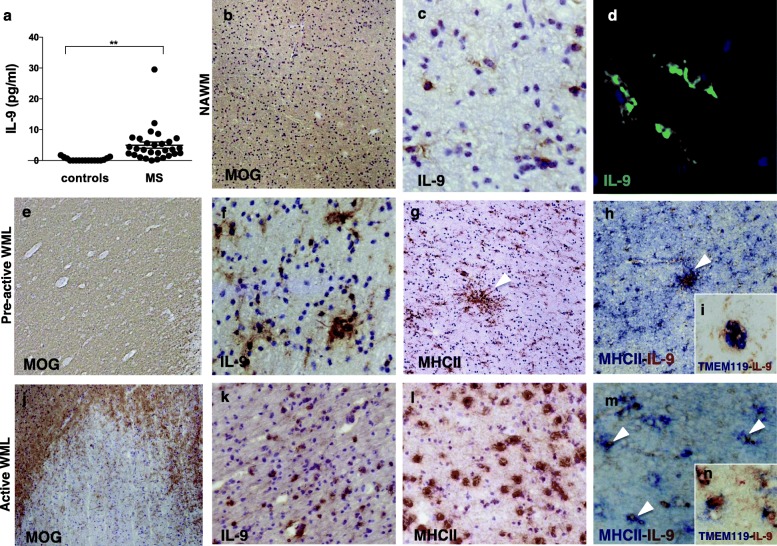
IL-9 is expressed in the central nervous system of secondary progressive MS patients. Molecular and neuropathological analysis of IL-9 expression in post-mortem MS white matter. CSF levels of IL-9 in the 29 post-mortem MS cases and 17 age-matched controls (**a**) (**p < 0.01). In normal appearing white matter (NAWM) identified by myelin oligodendrocyte glycoprotein (MOG) immunostaining (**b**), rare and scattered IL-9^+^ cells with microglia morphology (**c**, **d**) were detected by immunohistochemistry or immunofluorescence. In pre-active white matter lesions (WML) (**e**), where clusters of MHC class II^+^ microglia were present (arrowheads in **g**), increased expression of IL-9 was detected (**f**). Double immunohistochemistry demonstrated that most of the IL-9 was expressed by clusters of microglia expressing MHC-II (arrowheads in **h**) or TMEM-119 (**i**). In active WML, with on-going demyelination (**j**) and a high density of microglia/macrophages MHC-II^+^ (**l**), elevated IL-9 expression was observed (**k**), often in combination with MHC-II (arrowheads in **m**) or TMEM-119 (**n**). Original magnifications: × 100 (**b**, **e**, **g**, **h**, **l**, **k**), × 200 (**c**, **f**, **j**–**m**), ×400 (**d**, **i**, **n**)

To investigate the presence of IL-9 in the CNS of secondary progressive MS patients, IL-9 levels were measured in CSF obtained from post-mortem progressive MS patients and compared with levels measured in CSF from post-mortem non-neurological control subjects. Significantly higher levels of IL-9 (fold increase = 9; *p* < 0.01) were found in the CSF of MS patients compared to controls, indicating that IL-9 expression is upregulated in the CNS of progressive MS patients (Fig. 1a).

Using immunohistochemistry techniques, we analyzed post-mortem cerebral tissue from a subgroup of MS brains and non-neurological control brains, from which CSF samples were also analyzed. In 2 out of the 8 examined MS brain samples (Table 2; Fig. 1b–d), we found IL-9 expression in scattered cells in normal appearing (NA) white matter (WM). Comparing lesion types of WM according to their activity, we localized increased IL-9 expression mainly in pre-active (Fig. 1e–i) and in active lesions (Fig. 1j–n). IL-9 was also found to be expressed in some inactive lesions, however to a lesser extent (Table 2).

**Table 2:** Individual clinical, autopsy, and neuropathology details in white matter and gray matter of secondary progressive MS patients

Case		MS 92	MS 121	MS 160	MS 180	MS 234	MS 286	MS 342	MS 356	MS 389	MS 473
Sex/age at death (years)		F/38	F/49	F/44	F/44	F/39	M/45	F/35	F/45	F/55	F/39
Onset (years)		21	35	28	26	24	29	30	29	28	27
Disease duration (years)		17	14	16	18	15	16	5	16	27	12
Meningeal inflammation		+	–	+	+	–	+	–	–	–	–
Perivascular infiltration		+	+	–	–	+	+	+	–	+	–
IL-9^+^ in White matter	NAWM	–	+	–	–	–	+	–	–	–	–
	Pre-active	–	–	–	–	++	++	–	–	–	–
	Active	–	–	–	–	–	–	++	++	++	++
	Chronic active	+	+	–	–	–	+	–	–	–	–
	Remyelination	–	–	+	–	+	–	–	–	–	–
	Inactive	–	–	–	–	–	+	–	–	–	–
IL-9^+^ in Gray matter	NAGM	–	–	–	–	–	–	–	–	–	–
	Pre-active	–	–	–	–	–	–	–	–	–	–
	Active	–	–	+	–	+	+	–	–	–	–
	Chronic active	–	+	+	–	+	+	–	–	–	–
	Remyelination	–	–	–	–	–	–	–	–	–	–
	Inactive	–	–	–	–	+	–	–	–	–	–

The degree of meningeal and perivascular inflammation was scored as −, absent; +, present; and ++, abundant. Similarly, the presence of IL-9 expressing (IL-9^+^) cells in the different types of lesions was scored as: −, absent; +, present; and ++, abundant. Tissues are classified as follows: normal appearing white matter (NAWM), normal appearing gray matter (NAGM), pre-active, chronic active, remyelination, and inactive

**Fig. 2:**
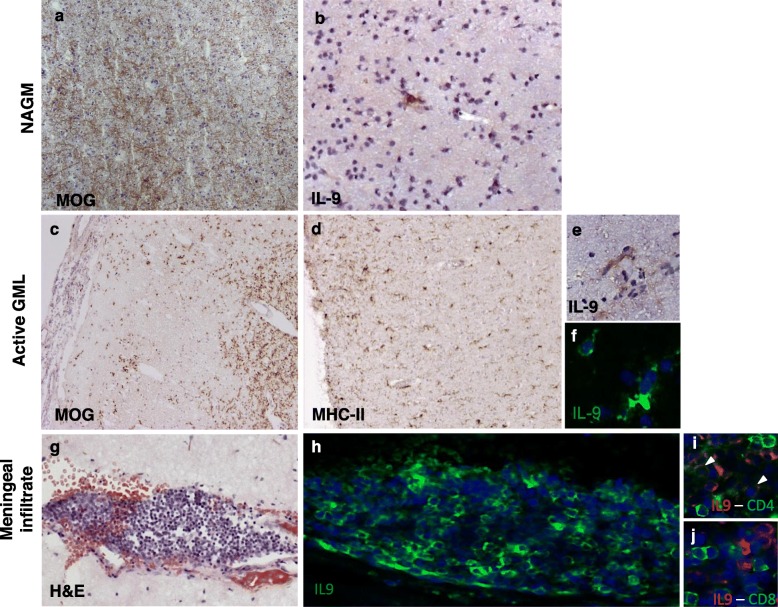
IL-9 is expressed in gray matter lesions and meninges of secondary progressive MS patients. Neuropathological analysis of IL-9 expression in post-mortem MS gray matter and meninges by immunohistochemistry and immunofluorescence. In normal appearing gray matter (NAGM) (**a**), rare IL-9^+^ cells with microglia morphology were detected (**b**). In active gray matter lesions (GML) (**c**), in areas containing MHC class II^+^ cells with microglia morphology (**d**), scattered IL-9^+^ were present (**e**, **f**), in particular in the external cortical layers close to the CSF surface or around small blood vessels. High numbers of IL-9^+^ cells (**h**) were observed in meningeal infiltrates (**g**) overlying the cortical gray matter. Double immunofluorescence for CD4 and IL-9 (**i**) or CD8 and IL-9 (**j**) of meningeal infiltrates in post-mortem brain tissues of progressive MS patients demonstrated that CD4^+^ (arrowheads in **i**), but not CD8^+^, T lymphocytes express IL-9. Original magnifications: × 100 (**a**, **c**, **d**), × 200 (**b**, **g**, **h**), × 400 (**e**, **f**, **i**, **j**)

In NAWM areas, IL-9 was expressed by groups of cells with the morphological characteristics of activated microglia (Fig. 1d). In pre-active and active WM lesions (WML) (Fig. 1e, j), a high density of IL-9^+^ cells was detected in areas of elevated MHC class II reactivity (Fig. 1f–h, k–m). A small proportion of IL-9^+^ cells were found to express TMEM119, a marker of resident microglia (Fig. 1i, n). Concerning gray matter (GM), scarce IL-9^+^ cells were detected in NAGM (Table 2; Fig. 2a, b) and more abundant IL-9^+^ cells in active and chronic active GML among the examined MS cases (Table 2; Fig. 2c–f). IL-9 was found to be expressed in cells with microglia morphology in active GML of 3 out of the 10 examined MS cases (Table 2, Fig. 2c–f).

Moreover, IL-9 was similarly expressed in cortical and subcortical areas of the same brain sections, as demonstrated by the immunostaining of active subpial and active leukocortical GM lesions (Supplementary Fig. 1).

IL-9 was found to be highly expressed by a proportion of meningeal inflammatory infiltrating cells (Fig. 2g, h) within the cerebral sulci adjacent to the GM. By using double immunofluorescence, IL-9^+^ cells in meningeal and perivascular infiltrate were identified as CD4 T lymphocytes (Fig. 2i), while none expressed CD8 (Fig. 2j), few IL9^+^ cells expressed the CD68, the marker of activated and macrophages (Supplementary Fig. 2).

### Among the immune cells, monocytes and macrophages expressed the highest levels of IL-9R

**Fig. 3:**
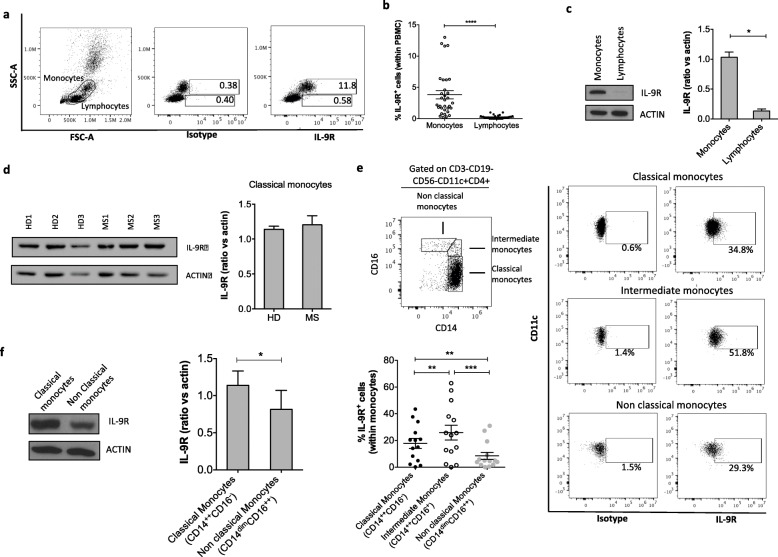
IL-9R is widely expressed by human monocytes in the blood. IL-9R expression by monocytes and lymphocytes within peripheral blood mononuclear cells (PBMC) of healthy donors was analyzed by flow cytometry. Plots from a representative experiment are shown (**a**). Graph represents the frequency of monocyte and lymphocyte IL-9R^+^ cells, gated on PBMC (**b**). IL-9R expression was analyzed by western blot on sorted monocytes and lymphocytes. Results from a representative donor and cumulative data of 3 donors are reported (**c**). IL-9R expression analyzed by western blot on sorted monocytes from MS patients and healthy donors. Results from three representative donors of each group and cumulative data are reported (**d**). IL-9R expression on classical (CD14^++^CD16^−^), intermediate (CD14^++^CD16^+^) and nonclassical (CD14^dim^CD16^++^) monocytes gated on CD3- CD19- CD56- CD11c+ CD4+ cells was analyzed by flow cytometry. Plots from a representative experiment and cumulative graph representing the frequency of monocyte subpopulation IL-9R^+^ cells (**e**). IL-9R expression was analyzed by western blot on sorted classical and nonclassical monocytes. Results from a representative donor and cumulative data of 4 donors are reported (**f**). Mean ± SEM is shown for each group. *p < 0.05, **p < 0.01, ***p < 0.001,****p < 0.0001 by paired Student’s *t* test (**b**, **c**, **d**, **f**) or by analysis of variance (**e**)

To identify which of the immune cells respond to IL-9, we systematically tested the expression of IL-9R on PBMCs obtained from healthy donors. We found the highest frequency of IL-9R^+^ cells in monocyte population, as demonstrated by flow cytometry (Fig. 3a, b) and validated by western blot (Fig. 3c). In contrast, fewer total lymphocytes (Fig. 3a–c), B lymphocytes, CD8 lymphocytes, mucosal associated invariant T cells (MAIT) cells, natural killer (NK) cells, T helper cells, and T regulatory cells (Supplementary Fig. 3A,B) express IL-9R. Importantly, monocytes from blood of MS patients express similar levels of IL-9R compared to those of healthy donors, indicating that monocytes may respond to IL-9 during MS disease (Fig. 3d).

Given the important distinction between “classical” monocytes (CD14^++^CD16^−^) that migrate to sites of injury where they differentiate into inflammatory macrophages [19], “intermediate” monocytes (CD14^++^CD16^+^) that possess inflammatory characteristics [20], and “nonclassical” monocytes (CD14^dim^CD16^++^) that exhibit a unique ability to patrol the resting vasculature and remove debris [21], we further characterized the expression of IL-9R on different classes of monocytes. We found that classical and intermediate monocytes are the immune cells most responsive to IL-9 (Fig. 3e, f).

**Fig. 4:**
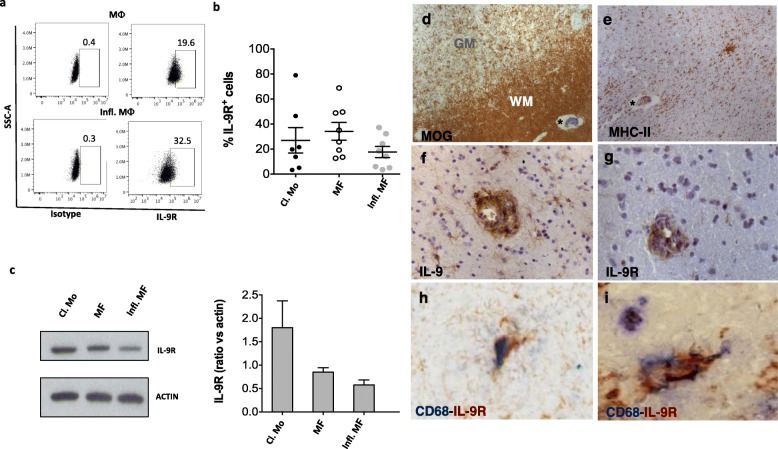
In vitro and in situ macrophages are responsive to IL-9. Human blood classical monocytes (Cl. Mo) of healthy donors were differentiated in macrophages (MΦ), and inflammatory macrophages (Infl. MΦ) in the presence of LPS and IFN-γ. IL-9R expression was analyzed by flow cytometry. Plots from a representative experiment are shown (**a**). Graph represents the frequency of IL-9R^+^ cells (**b**). IL-9R expression was analyzed by western blot. Results from a representative donor and cumulative data of 4 donors are reported (**c**). Mean ± SEM is shown for each group. Neuropathological assessment of IL-9 and IL-9R expression in post-mortem MS brain. Areas of microglia activation (**e**) in white matter (**d**), indicated with an asterisk, contain IL-9^+^ and IL-9R^+^ cells, mainly expressed in perivascular infiltrates (**f**, **g**). Scattered CD68^+^ IL-9R^+^ cells were found in the white matter tissue (**h**, **i**). Original magnifications: × 100 (**d**, **e**), × 200 (**f**, **g**), × 400 (**h**, **i**)

Moreover, myeloid dendritic cells express higher levels of IL-9R than plasmacytoid dendritic cells (Supplementary Fig. 4A, B). Next, we analyzed the expression of IL-9R in monocyte-derived human macrophages. We found that similar to freshly purified monocytes, all macrophage subtypes express IL-9R (Fig. 4a–c).

### IL-9R is expressed by macrophages in active MS lesions

In order to investigate the IL-9 responsiveness of resident CNS immune cells in MS, we analyzed immunohistochemistry staining of IL-9R in post-mortem brain tissues of MS patients. We found most of the IL-9R^+^ cells (Fig. 4g) in perivascular inflammatory infiltrates, mainly in the white matter (Fig. 4d), in the presence of diffuse microglia/macrophage activation (Fig. 4e) and in areas containing IL-9^+^ cells (Fig. 4f). Double immunofluorescence revealed that IL-9R is expressed by some CD68^+^ macrophages/microglia in CNS (Fig. 4h, i) but not by CD3^+^ T cells or CD20^+^ B cells (data not shown).

### IL-9 reduces activation of human macrophages

Considering the responsiveness of macrophages to IL-9 and their relevance in the context of MS, we set to examine how IL-9 affects macrophages.

**Fig. 5:**
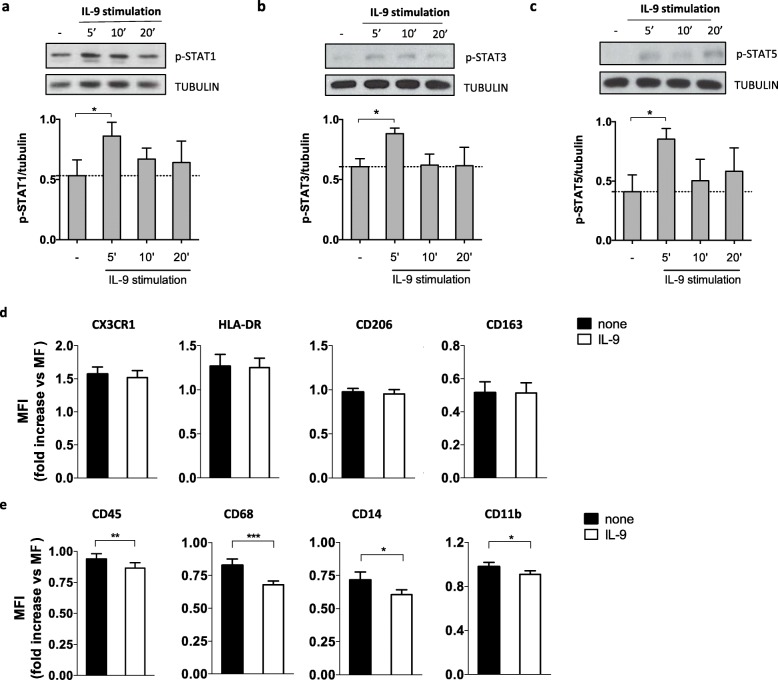
IL-9 activates STATs and reduces inflammatory properties in human macrophages. Human macrophages differentiated in vitro from blood classical monocytes were stimulated with IL-9 (200 ng/ml) for 5, 10, and 20 min and compared to unstimulated cells. The phosphorylation of STAT1 (**a**), STAT3 (**b**), and STAT5 (**c**) was analyzed by western blot. Results from a representative donor and cumulative data of 3 donors are reported. Mean ± SEM is shown for each group. *p < 0.05, by paired Student’s *t* test. Human macrophages differentiated as inflammatory macrophages (Infl. MΦ) from blood classical monocytes of healthy donors (*n* = 18) were pretreated with IL-9 for 24 h and treated with specific polarizing conditions for 48 h. The expression of CX3CR1, HLA-DR, CD206, CD163 (**d**), and CD45, CD68, CD14, CD11b (**e**) was analyzed by flow cytometry. Data are reported as fold change with respect to macrophages (MΦ). Paired Student’s *t* test was used to compare different conditions. *p < 0.05; **p < 0.01;***p < 0.001

In particular, we mimic the IL-9 stimulation that resident or infiltrating macrophages receive in the CNS by using an in vitro model of human macrophages differentiated from blood monocytes of healthy donors, and stimulation with recombinant IL-9. Then, we measured the resulting downstream phosphorylation of STAT1, 3, and 5. We found that IL-9 induced phosphorylation of STAT1, 3, and 5, with peak activation after 5 min (Fig. 5a–c). Next, we used IFN-γ + LPS to obtain inflammatory macrophages (Fig. 4a, c), and we sought to determine whether IL-9 affects the activation of macrophages. To this end, we analyzed the expression of macrophages’ activation markers on the cell surface as well as the cytokines released in their supernatants upon in vitro stimulation with exogenous recombinant IL-9. Although typical markers of the pro-inflammatory or anti-inflammatory profiles, such as CX3CR1 and HLA-DR or CD206 and CD163, respectively, were not modulated by IL-9 (Fig. 5d), IL-9 reduced inflammatory properties of inflammatory macrophages, decreasing the expression of activation markers, such as CD45 (7.9% ± 2.3), CD11b (7.2% ± 2.4), CD68 (16,5% ± 3.3), and CD14 (13.3% ± 3.6), reported here as percentage decrease (mean ± SEM) of the mean fluorescence intensity (MFI) (Fig. 5e).

**Fig. 6:**
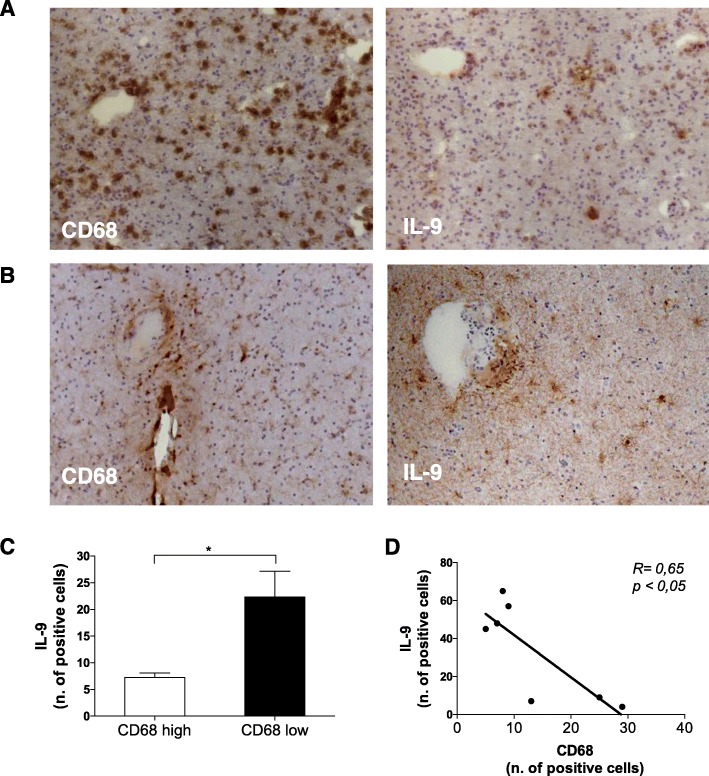
IL-9 regulates macrophage activation in the brain of secondary progressive MS patients. Adjacent frozen sections of post-mortem brain of progressive MS patients were stained with anti-CD68 and anti-IL-9 (1 representative of “CD68 high” group in **a**; 1 representative of “CD68 low” group in **b**). Counts of total positive cells for IL-9 in brain biopsy specimens from 3 patients of “CD68 high” and 4 patients with “CD68 low” group were compared. Mean ± SEM are reported (**c**). *p < 0.05. The count of IL-9 positive cells inversely correlated to CD68 positive cells with the Pearson correlation (*R*, correlation coefficient)

In order to analyze whether IL-9 expression is related somehow to macrophage activation also in situ, we stained 7 post-mortem MS brain tissues for the macrophage activation marker CD68 and for IL-9. By grouping MS patients in group 1 characterized by high CD68 expression, and group 2 characterized by low CD68 expression, we found that patients in group 1 express low levels of IL-9 (Fig. 6a), while patients in group 2 express high levels of IL-9 (Fig. 6b). We found that the number of IL-9 positive cells in group 1 and group 2 were significantly different (Fig. 6c) and that the number of IL-9^+^ cells inversely correlates with number of CD68 positive cells in the same brain tissue (Fig. 6d). The two groups do not differ for any clinical and demographic parameter. However, the tissue blocks of CD68-high MS cases examined in the current study present higher number of active lesions with respect to CD68-low supporting the elevated degree of active demyelination in the subgroup of CD68-high and IL-9-low MS cases.

### IL-9 has an anti-inflammatory effect on human macrophages

Although macrophages and inflammatory macrophages (obtained with LPS + IFN-γ) do not produce detectable levels of IL-9 (< 1.5 pg/ml), we investigated whether the production of other cytokines was modulated by IL-9 stimulation.

**Fig. 7:**
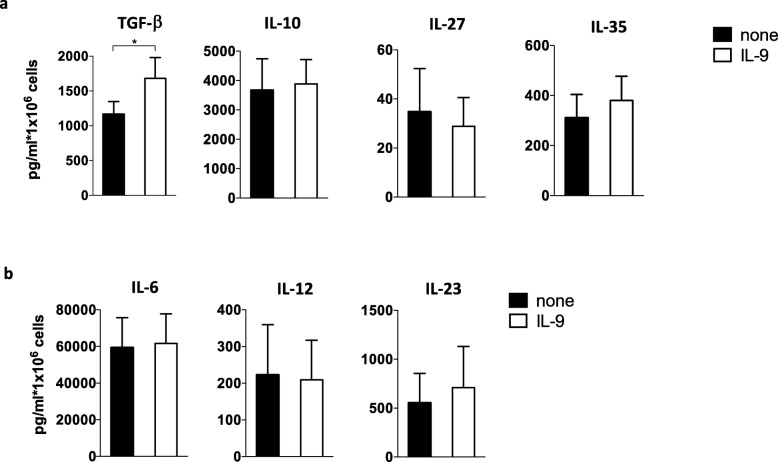
IL-9 induces TGF-β production by human macrophages. TGF-β1, IL-10, IL-27, IL-35 (**a**), and IL-6, IL-12, IL-23 (**b**) production was analyzed in the supernatants by ELISA. Paired Student’s *t* test was used to compare different conditions. *p < 0.05

We analyzed the anti-inflammatory cytokines TGF-β1, by human macrophages stimulated with IL-9. We found that TGF-β1 production was significantly increased (67% ± 24; percentage increase pg/ml mean ± SEM), while the production of IL-10, IL-27, and IL-35 was not affected by IL-9 (Fig. 7a).

In contrast, the pro-inflammatory cytokines IL-6, IL-12, and IL-23 were not modulated by IL-9 (Fig. 7b). These results are in line with a protective role of IL-9 in MS and suggest that, at least in part, this protection is mediated by an IL-9-induced reduction of macrophage inflammatory properties and increase of TGF-β release.

## Discussion

In MS, resident and infiltrating macrophages accumulate in the brain and can exert both pro- or anti-inflammatory activities or direct phagocytic myelin-damaging activity [22], according to the pathological and inflammatory conditions.

Our study here reveals that IL-9 is expressed in the brain of progressive MS patients, that microglia and macrophages respond to IL-9 in the brain, and that IL-9 regulates the functional properties of human macrophages, shifting their phenotype toward an anti-inflammatory profile.

The down-regulation of typical activation markers, such as CD11b, CD14, CD68, and CD45, known to mediate important processes such as adhesion to endothelium, migration, and phagocytosis [23–25], indicates that IL-9 may reduce and regulate infiltration and phagocytic activity of macrophages. Furthermore, we observed an induction of the anti-inflammatory cytokine TGF-β1, which is in line with the effect previously observed in LPS-stimulated monocytes [9] and alveolar macrophages [8]. Interestingly, in murine mast cells, the release of mast cell protease-1 induced by IL-9 depends on TGF-β, suggesting that mast cells may also produce TGF-β upon IL-9 treatment [26]. This anti-inflammatory function of IL-9 on macrophages could be relevant for the damping of exaggerated inflammation, observed in autoimmune diseases and even serve as target for future therapeutic avenues. In particular, the IL-9 expression observed in cortical lesions, both subpial and leukocortical, may suggest a key role of IL-9 in regulating the inflammatory events involved in neurodegeneration.

We previously reported that high levels of IL-9 in CSF of RR-MS patients during the MS diagnosis are associated with lower disease progression over 4 years of clinical follow-up [5]. Overall, these results support the hypothesis that an intrinsic high expression of IL-9, before the disease establishment, correlates with a better clinical profile of MS patients suggesting IL-9 as a prognostic biomarker of better outcome of the disease. Even if these data need to be validated in larger and independent MS cohorts, they are supported by recent data showing that high IL-9 gene expression in the meninges is associated with a subgroup of post-mortem progressive MS patients with low levels of meningeal inflammation and cortical demyelination, and a less severe and rapid disease course [16]. Here, we report the presence of IL-9 protein in both CSF and CNS, particularly in pre-active and active lesions, of the same examined post-mortem progressive MS patients that indicates a consistent potential beneficial effect of IL-9 not only in RR but also in progressive MS disease. These data highlight the need to better investigate and validate the potential value of CSF levels of IL-9, which could be then used as prognostic biomarker of a specific MS endophenotype with better disease outcome and as marker to monitor responses to therapies.

Given the IL-9 expression mainly associated to macrophages and activated microglia in WM and GM lesions, these cells may represent the main cell sources of IL-9 found in the CSF of both RR- [5] and progressive MS patients. However, the stimuli triggering IL-9 production by microglia/macrophages *in vivo* and in vitro are not known. The fact that IL-9 expression seems to be present both at time of diagnosis and during the disease progression would indicate its possible involvement in chronic inflammation characterizing MS. Further analysis of the specific molecular characterization of the tissue resident and peripheral cells expressing IL-9, and of the interacting inflammatory conditions/milieu, would help to better understand potential useful therapeutic targets.

In meningeal infiltrates of post-mortem progressive MS patients, IL-9 is also expressed by CD4^+^ T lymphocytes. The latter corroborate previous findings that a specific subpopulation of CD4^+^ T lymphocytes, named Th9 cells, are characterized by IL-9 production [27], in response to a complex cytokine milieu. In fact, it is known that in vitro lymphocytes, natural killer cells, and mast cells [28] produce IL-9, particularly following exposure to TGF-β and IL-4 [29]. However, we previously reported that in vitro production of IL-9 by Th9 cells obtained from CD4+ T lymphocytes stimulated with TGF-β and IL-4 is similar in MS and healthy donors [5], indicating that environmental conditions may influence the *in vivo* induction of IL-9 by lymphocytes in the brain of MS patients. In addition, our data help to characterize inflammatory features of CD4^+^ T cells in meningeal infiltrates, suggesting that this cytokine may be involved in intrathecal compartmentalization of MS inflammation, in particular in the subarachnoid space.

Several costimulatory signals provided by other inflammatory and antigen-presenting cells within the inflammatory infiltrates, including the meningeal ones, may affect the release and abundance of IL-9 in the CNS. In this context, others and we previously demonstrated that OX40-OX40-L interaction between antigen-presenting cells and T cells could be involved in IL-9 induction [30, 31].

Further investigation of factors modulating IL-9 induction during MS, are needed. Moreover, IL-9 expression may be influenced also by genetic and epigenetic modifications, which might explain the diversity of expression levels among individuals, and correlate with the different clinical outcomes of MS patients.

The functions related to IL-9 are mediated by its specific receptor, IL-9R, known to be expressed on Th17 and Treg cells, monocytes, and mast cells. Here, we demonstrate that macrophages of post-mortem MS tissues express IL-9R in the same inflammatory infiltrates in which IL-9 is expressed. Moreover, our detailed and systematic analysis of IL-9R expression on human blood immune cells revealed that classical monocytes (CD14^++^CD16^−^) and intermediate monocytes (CD14^++^CD16^+^) express highest levels of IL-9R, suggesting them as potential source of macrophages infiltrated in the CNS and responsive to IL-9.

In contrast, nonclassical monocytes (CD14^dim^ CD16^+^), which are patrolling monocytes with a high ability to migrate across endothelial monolayers [32] and to remove damaged cells and debris from the vasculature [21], express lower levels of IL-9R compared to other monocytes. Similarly, plasmacytoid dendritic cells, known for their induction of IL-10 producing T regulatory cells [33, 34], express lower levels of IL-9R, compared to myeloid dendritic cells. These results, together with the specificity of IL-9 effects on pro-inflammatory macrophages, suggest that cells committed to resolve inflammation are less responsive to IL-9, likely due to an IL-9-mediated feedback control on inflammatory cells. Considering the key role of macrophages in phagocytosis, antigen presentation and lymphocyte stimulation, the orchestrated expression of IL-9 and its receptor in MS patients may facilitate a specific immune response that ameliorates the clinical outcome.

Finally, these results highlight a new mechanism regulating progressive MS disease and suggest either a potential good biomarker of disease inflammatory activity or a novel approach for therapeutic intervention.

Moreover, the regulatory role played by IL-9 in human inflammatory macrophages within the CNS, could be relevant in other CNS disorders, including neurodegenerative diseases, such as Alzheimer, Parkinson, and amyotrophic lateral sclerosis diseases [35], where even if the pathological processes are not primarily inflammatory driven, mononuclear phagocytes are known to contribute to the development of pathology.

## Conclusions

IL-9 is a new cytokine expressed by microglia and CD4^+^ T cells in lesions localized in both gray and white matter, as well as in inflamed meninges of MS patients, and its receptor, IL-9R, is expressed by macrophages/microglia in the areas IL-9^+^ of the CNS. We found that IL-9 downregulates macrophage activation via phosphorylation of STAT1, 3, and 5. This is a new immune mechanism that could regulate inflammation in CNS of progressive MS patients. Given the absence of a therapy for the progressive forms of MS and the unclear knowledge of the mechanisms leading to the pathology, these findings could have important clinical therapeutic implications.

## Supplementary information

**Additional file 1: Figure S1.** IL-9 is expressed in subpial and leukortical gray matter lesions of progressive MS cases. Active subpial gray matter lesion (GML) (A-C) and active leukocortical GML (D-F) of post-mortem brain tissues of progressive MS patients were stained for myelin oligodendrocyte glycoprotein (MOG) (A,D), and IL-9 and analyzed by immunohistochemistry (B,E) or immunofluorescence (C,F). Original magnifications: 100x (A,D), 200x (B, E), 400x (C,F). **Figure S2.** Macrophages infiltrating the brain of secondary progressive MS patients express IL-9. Double immunohistochemistry for CD68 and IL-9 in post-mortem brain tissues of progressive MS patients demonstrates that some CD68^+^ macrophages express IL-9 (A). Original magnifications: 100x (A), 400x (B). **Figure S3.** Human lymphocytes express low levels of IL-9R in the blood. IL-9R expression on B lymphocytes, CD8+ lymphocytes, MAIT cells, NK cells, T regulatory cells (A) and on T helper (Th) 1, Th1/Th17, Th17 and Th2 subsets was analyzed by flow cytometry. Graph represents the frequency of lymphocyte subpopulation IL-9R^+^ cells (A, B). Mean ± SEM is shown for each group. **Figure S4.** Myeloid dendritic cells express higher levels of IL-9R compared to plasmacytoid dendritic cells in the blood. IL-9R expression on plasmacytoid myeloid (CD4 + CD11c-) and myeloid (CD4 + CD11c+) dendritic cells (pDC and mDC, respectively) gated on CD3-CD19-CD56-CD14-CD16- cells of healthy donors’ PBMC was analyzed by flow cytometry. Graph represents the frequency of IL-9R+ pDC and mDC cells (A). IL-9R expression was analyzed by Western blot on sorted pDC and mDC. Results from a representative donor and cumulative data of 6 donors are reported (B). Mean ± SEM is shown for each group.*p < 0.05.

## Data Availability

Not applicable

## Abbreviations

MS: Multiple sclerosis
CNS: Central nervous system
IL: Interleukin
STAT: Signal transducer and activator of transcription
IFN: Interferon
TGF: Transforming growth factor
CSF: Cerebrospinal fluid
RR: Relapsing remitting
JAK: Janus kinase
EAE: Experimental autoimmune encephalomyelitis
MOG: Myelin oligodendrocyte glycoprotein
MHC: Major histocompatibility complex
IHC: Immunohistochemistry
IF: Immunofluorescence
PBMC: Peripheral blood mononuclear cells
FBS: Fetal bovine serum
M-CSF: Macrophage colony-stimulating factor
LPS: Lipopolysaccharides
ANOVA: Analysis of variance
NAWM: Normal appearing white matter
WML: White matter lesion
GML: Gray matter lesion
MAIT: Mucosal associated invariant T cells
NK: Natural killer
MFI: Mean fluorescence intensity
