# Supplementary material for: Interleukin-9 regulates macrophage activation in the progressive multiple sclerosis brain
Source: J Neuroinflammation. 2020 May 6;17:149. doi: 10.1186/s12974-020-01770-z (PMC7204302; doi:10.1186/s12974-020-01770-z)
Supplement: Supplementary file 1 — Additional file 1: Figure S1. IL-9 is expressed in subpial and leukortical gray matter lesions of progressive MS cases. Active subpial gray matter lesion (GML) (A-C) and active leukocortical GML (D-F) of post-mortem brain tissues of progressive MS patients were stained for myelin oligodendrocyte glycoprotein (MOG) (A,D), and IL-9 and analyzed by immunohistochemistry (B,E) or immunofluorescence (C,F). Original magnifications: 100x (A,D), 200x (B, E), 400x (C,F). Figure S2. Macrophages infiltrating the brain of secondary progressive MS patients express IL-9. Double immunohistochemistry for CD68 and IL-9 in post-mortem brain tissues of progressive MS patients demonstrates that some CD68+ macrophages express IL-9 (A). Original magnifications: 100x (A), 400x (B). Figure S3. Human lymphocytes express low levels of IL-9R in the blood. IL-9R expression on B lymphocytes, CD8+ lymphocytes, MAIT cells, NK cells, T regulatory cells (A) and on T helper (Th) 1, Th1/Th17, Th17 and Th2 subsets was analyzed by flow cytometry. Graph represents the frequency of lymphocyte subpopulation IL-9R+ cells (A, B). Mean ± SEM is shown for each group. Figure S4. Myeloid dendritic cells express higher levels of IL-9R compared to plasmacytoid dendritic cells in the blood. IL-9R expression on plasmacytoid myeloid (CD4 + CD11c-) and myeloid (CD4 + CD11c+) dendritic cells (pDC and mDC, respectively) gated on CD3-CD19-CD56-CD14-CD16- cells of healthy donors’ PBMC was analyzed by flow cytometry. Graph represents the frequency of IL-9R+ pDC and mDC cells (A). IL-9R expression was analyzed by Western blot on sorted pDC and mDC. Results from a representative donor and cumulative data of 6 donors are reported (B). Mean ± SEM is shown for each group.*p < 0.05. [file 12974_2020_1770_MOESM1_ESM.pptx]

## Slide 1
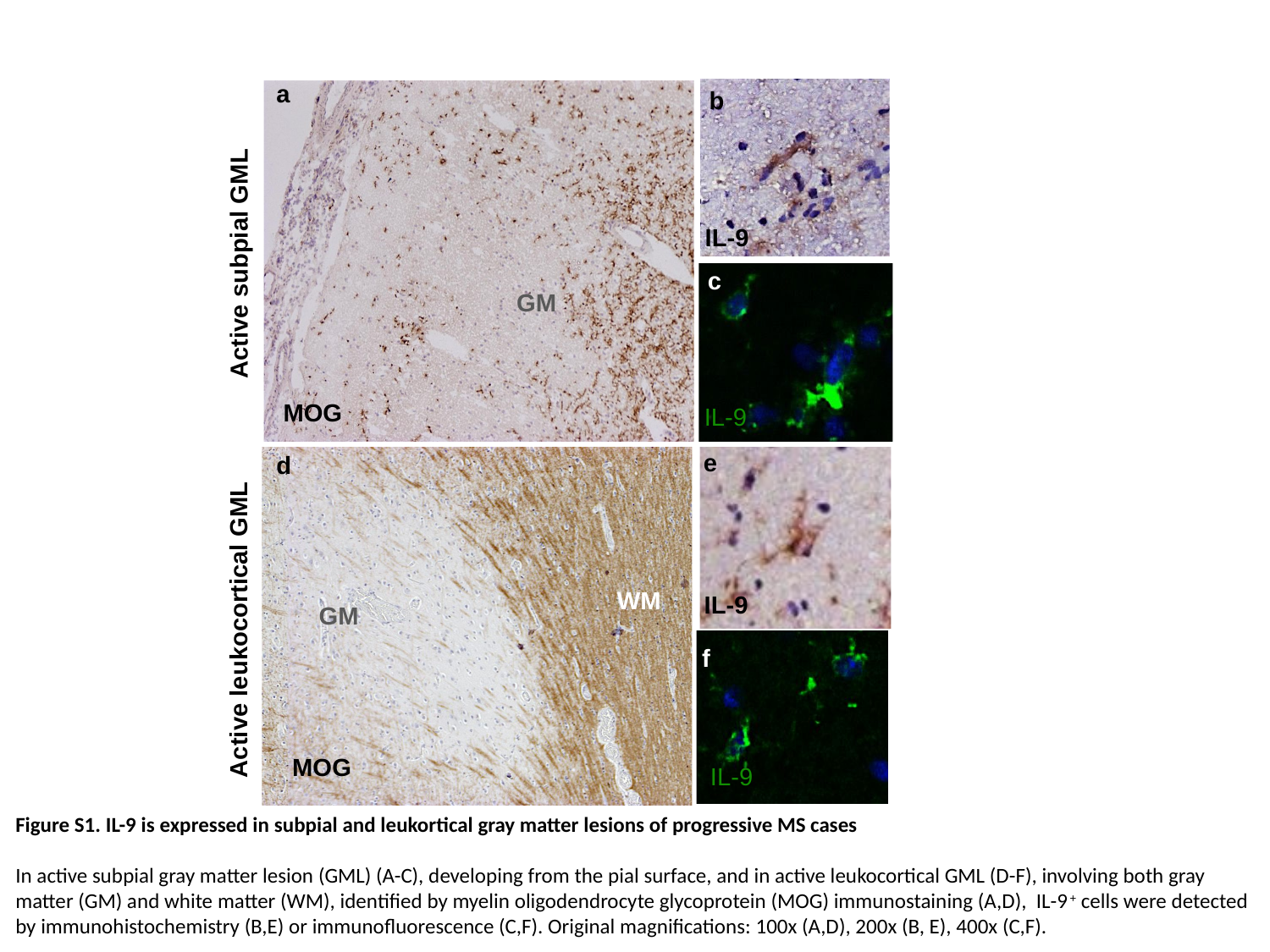

a
b
Active subpial GML
IL-9
c
 GM
 MOG
IL-9
e
d
Active leukocortical GML
 WM
IL-9
 GM
f
 MOG
IL-9
J
Figure S1. IL-9 is expressed in subpial and leukortical gray matter lesions of progressive MS cases
In active subpial gray matter lesion (GML) (A-C), developing from the pial surface, and in active leukocortical GML (D-F), involving both gray matter (GM) and white matter (WM), identified by myelin oligodendrocyte glycoprotein (MOG) immunostaining (A,D), IL-9+ cells were detected by immunohistochemistry (B,E) or immunofluorescence (C,F). Original magnifications: 100x (A,D), 200x (B, E), 400x (C,F).

## Slide 2
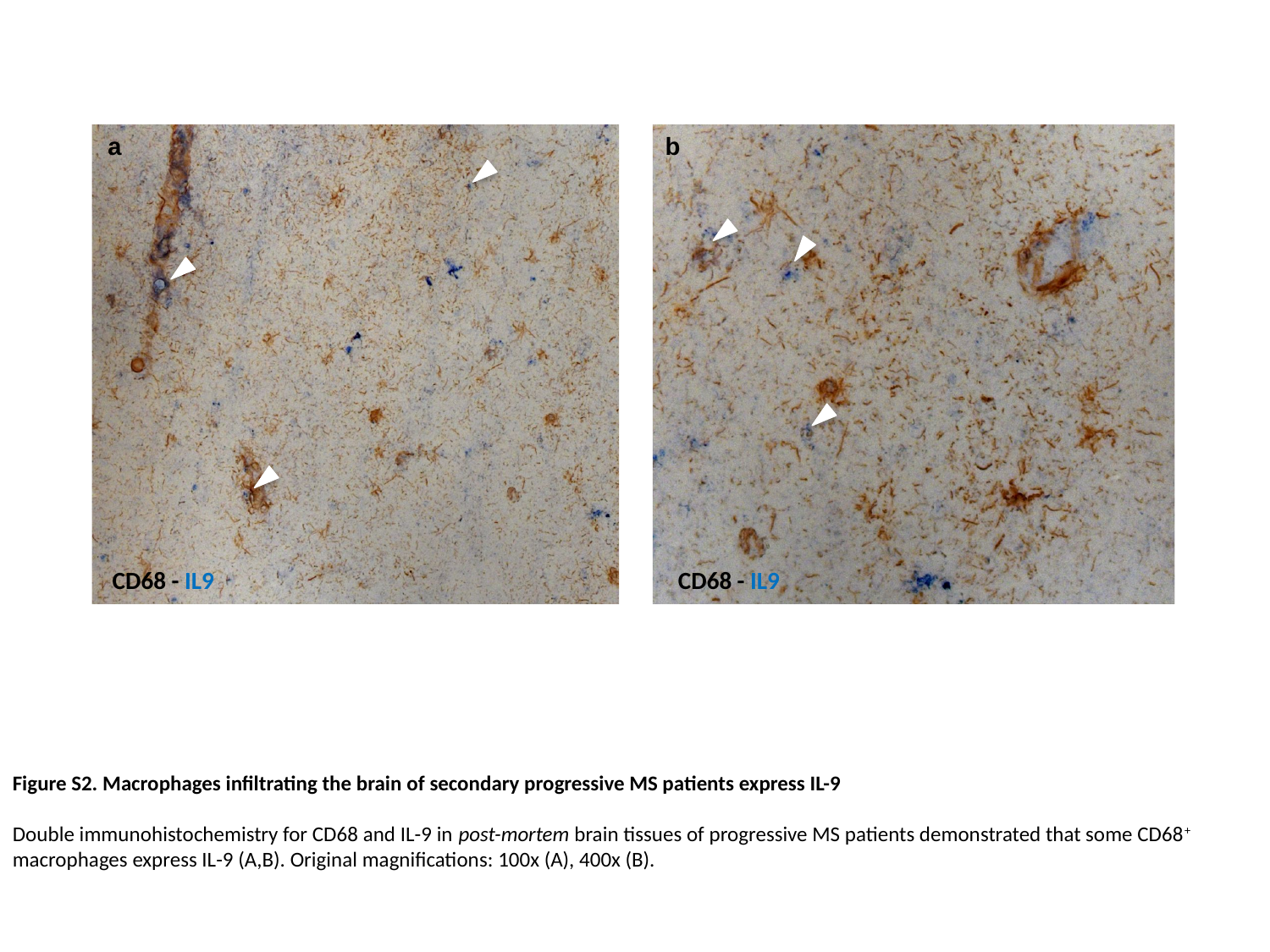

a
b
CD68 - IL9
CD68 - IL9
Figure S2. Macrophages infiltrating the brain of secondary progressive MS patients express IL-9
Double immunohistochemistry for CD68 and IL-9 in post-mortem brain tissues of progressive MS patients demonstrated that some CD68+ macrophages express IL-9 (A,B). Original magnifications: 100x (A), 400x (B).

## Slide 3
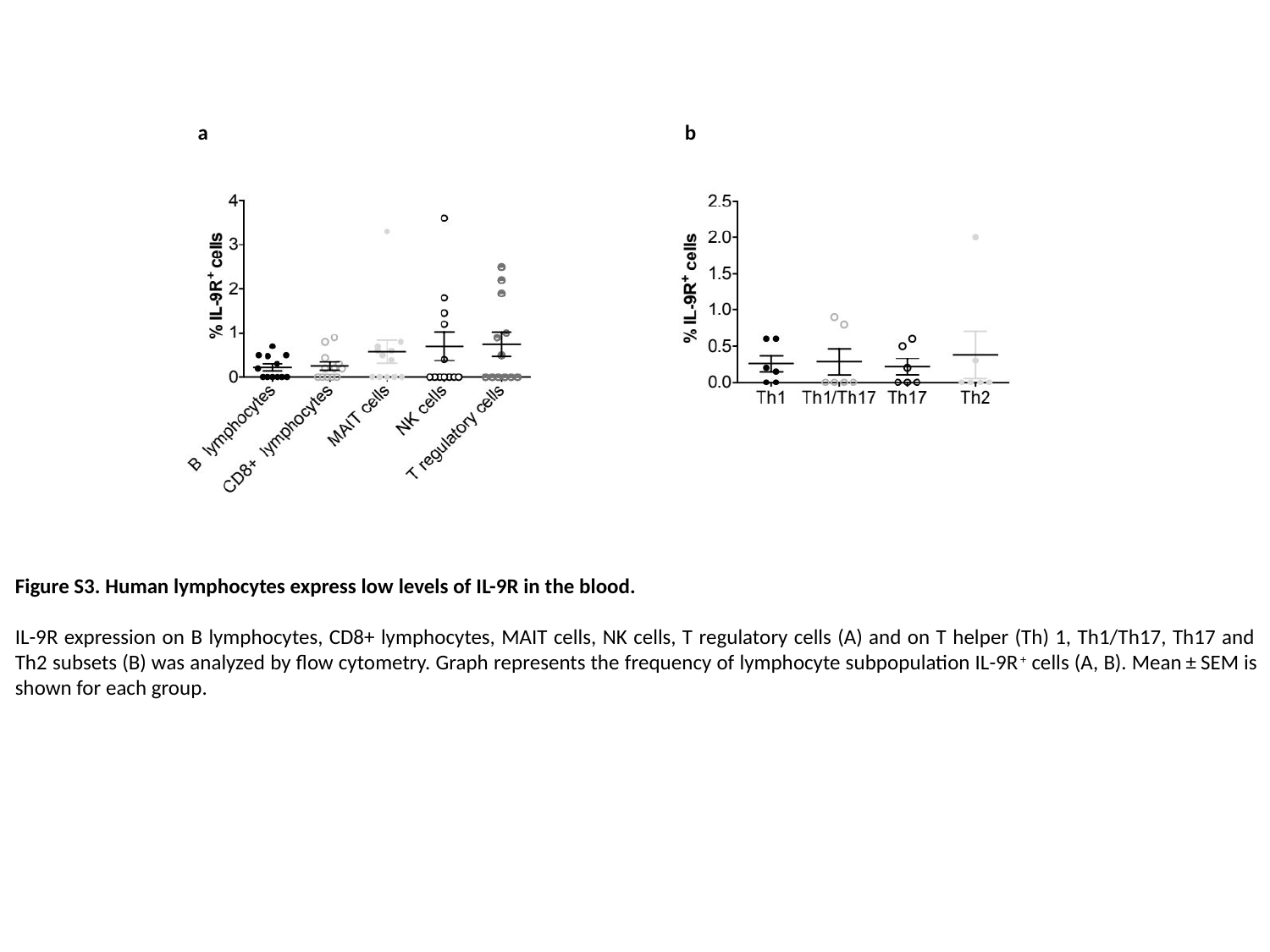

a
b
Figure S3. Human lymphocytes express low levels of IL-9R in the blood.
IL-9R expression on B lymphocytes, CD8+ lymphocytes, MAIT cells, NK cells, T regulatory cells (A) and on T helper (Th) 1, Th1/Th17, Th17 and Th2 subsets (B) was analyzed by flow cytometry. Graph represents the frequency of lymphocyte subpopulation IL-9R+ cells (A, B). Mean ± SEM is shown for each group.

## Slide 4
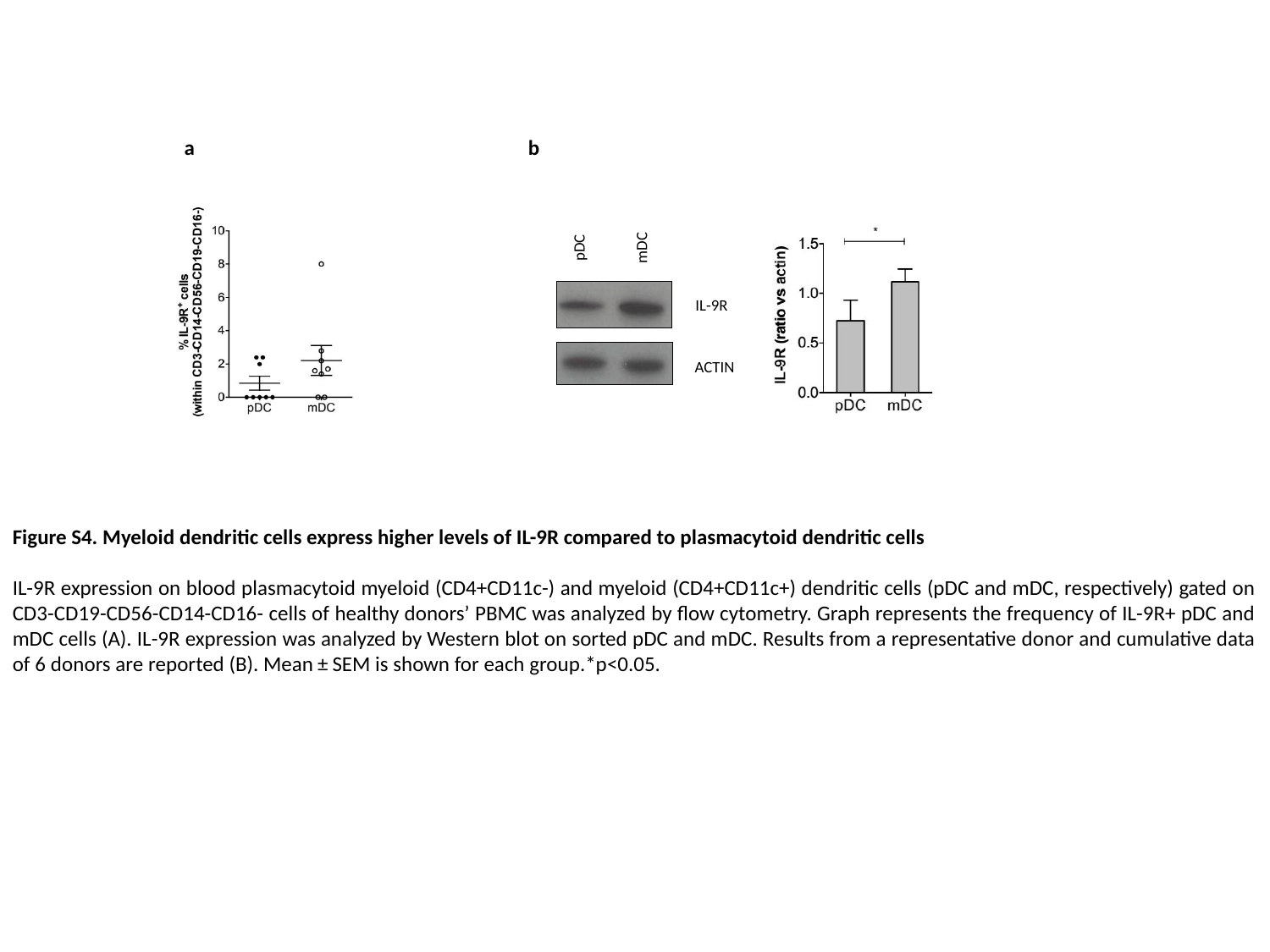

a
b
pDC
mDC
IL-9R
ACTIN
Figure S4. Myeloid dendritic cells express higher levels of IL-9R compared to plasmacytoid dendritic cells
IL-9R expression on blood plasmacytoid myeloid (CD4+CD11c-) and myeloid (CD4+CD11c+) dendritic cells (pDC and mDC, respectively) gated on CD3-CD19-CD56-CD14-CD16- cells of healthy donors’ PBMC was analyzed by flow cytometry. Graph represents the frequency of IL-9R+ pDC and mDC cells (A). IL-9R expression was analyzed by Western blot on sorted pDC and mDC. Results from a representative donor and cumulative data of 6 donors are reported (B). Mean ± SEM is shown for each group.*p<0.05.
